# Nonvolatile electric-field control of magnetization in a Y-type hexaferrite

**DOI:** 10.1038/srep08254

**Published:** 2015-02-05

**Authors:** Shipeng Shen, Yisheng Chai, Young Sun

**Affiliations:** 1 Beijing National Laboratory for Condensed Matter Physics, Institute of Physics, Chinese Academy of Sciences, Beijing 100190, China

## Abstract

The magnetoelectric effects in multiferroic materials enable the mutual control of electric polarization by a magnetic field and magnetization by an electric field. Nonvolatile electric-field control of magnetization is extremely important for information storage applications, but has been rarely realized in single-phase multiferroic materials. Here we demonstrate the prominent direct and converse magnetoelectric effects in the Y-type hexaferrite BaSrCoZnFe_11_AlO_22_ single crystal. The electric polarization due to conical magnetic structure can be totally reversed by a small magnetic field, giving rise to large magnetoelectric coefficients of 6000 and 4000 ps/m at 100 and 200 K, respectively. The *ab*-plane magnetization can be controlled by electric fields with a large hysteresis, leading to nonvolatile change of magnetization. In addition, the reversal of magnetization by electric fields is also realized at 200 K. These diverse magnetoelectric effects with large coefficients highlight the promise of hexaferrites as potential multiferroic materials.

Electric-field (E) control of magnetism has aroused intense interests due to its potential to develop new spintronic and electronic devices^1^,^2^,^3^. Many schemes have been attempted in the past decade to achieve this goal. In heterostructures and thin films, magnetization can be electrically modified via strain from piezoelectric substrates^4^,^5^,^6^,^7^,^8^,^9^; via exchange bias from antiferromagnetic-ferroelectric (BiFeO_3_)^10^,^11^,^12^ or magnetoelectric (Cr_2_O_3_) substrates^13^,^14^ *etc*. For the case of single-phase materials, magnetoelectrics and magnetoelectic (ME) multiferroics are top candidates to achieve the direct *E* control of magnetizations (*M*) because of its intrinsic ME coupling^15^,^16^,^17^,^18^,^19^. However, such a converse ME effect usually requires a large external magnetic field (H) or low temperature, which is not practical for device application. Furthermore, nonvolatile *E* control of magnetization, which is important for information storage, poses more severe challenges for ME multiferroics.

Hexaferrites with conical magnetic structures are promising multiferroic materials which show direct ME effect (the manipulation of polarization *P* by *H*) up to room temperature^20^,^21^,^22^,^23^,^24^. The converse ME effect was also observed in several hexaferrites^25^,^26^,^27^,^28^. For instance, *E* control of *M* was realized in Ba_0.5_Sr_1.5_Zn_2_(Fe_0.92_Al_0.08_)_12_O_22_ single crystal below 170 K. Even at room temperature, converse ME effects were still observed in Y- and Z-type hexaferrites ceramics. Nevertheless, the reported converse ME effects are usually volatile - after removing *E* field, *M* restores the initial state. Although nonvolatile *E* control of *M* was recently reported in Y-type hexaferrite ceramics^27^, the hysteresis of the *M-E* curve is very small, with a tiny remanent magnetization in zero *E* field, which is unsuitable for nonvolatile applications. In this work, we performed a systematic study on both the direct and converse ME effects in the Y-type hexaferrite BaSrCoZnFe_11_AlO_22_ single crystal. The results demonstrate pronounced ME effects with the direct ME coefficients of 6000 and 4000 ps/m at 100 and 200 K, respectively. Moreover, the converse ME effect exhibits a large hysteresis in the *M* – *E* loop which enables a clear nonvolatile *E* control of *M*.

## Results

### Characterization of the Y-type hexaferrite BaSrCoZnFe_11_AlO_22_

**Figure 1:**
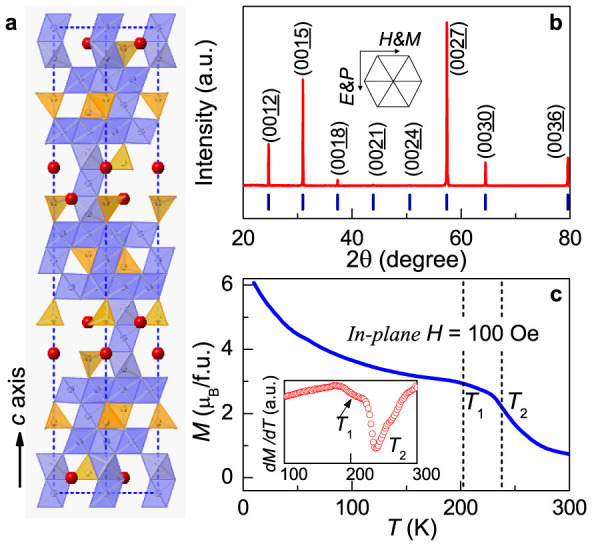
Characterization of the Y-type hexaferrite BaSrCoZnFe_11_AlO_22_. (a) The schematic crystal structure of Y-type hexaferrite. (b) The X-ray diffraction pattern of the Y-type hexaferrite single crystal sample along [001] direction. Inset of panel (b) shows the schematic experimental configuration. (c) Temperature dependent magnetization with *H* = 100 Oe along [100] axis. Before the measurements, *H* = 10 kOe was applied at 10 K to induce a metastable commensurate transverse cone state, then *H* was ramped down to 100 Oe. The Inset shows the derivative d*M*/d*T* as a function of temperature.

As shown in Fig. 1a, the Y-type hexaferrite has a stacked layer structure with the space group *R*-3m. Our prepared single crystals of BaSrCoZnFe_11_AlO_22_ were characterized by single crystal x-ray diffraction (XRD), and the room-temperature XRD pattern (Fig. 1b) suggests that our specimen belongs to Y-type hexaferrite with *c* = 4.32 nm.

Fig. 1c shows the temperature (*T*) dependent magnetization curve with *H* = 100 Oe along the [100] direction. Before the measurement, an external magnetic field *H* = 10 kOe was first applied at 10 K to induce commensurate transverse cone state^29^, which is an intermediate ferroelectric (FE) phase according to the spin-current (KNB) or inverse Dzyaloshinskii-Moriya (DM) interaction models^30^,^31^; then *H* was ramped down to 100 Oe. The commensurate transverse cone would be kept under *H* = 100 Oe. Two critical temperatures *T*_1_ and *T*_2_ are observed in the *M* – *T* and d*M*/d*T* curves (Fig. 1c). At *T*_1_ ~ 200 K the magnetization starts to drop more abruptly, suggesting that the commensurate transverse cone is no longer stable near zero magnetic field. The magnetodielelectric and magnetoelectric current measurements, as discussed in next section, also suggest that the commensurate transverse cone is likely to change to the longitudinal cone above *T*_1_ due to the change of magnetic anisotropy. With further increasing temperature, there is another critical temperature *T*_2_ and it is likely to be the transition to proper screw due to its easy in-plane anisotropy at higher temperatures^32^.

### Magnetic field control of electric polarization

**Figure 2:**
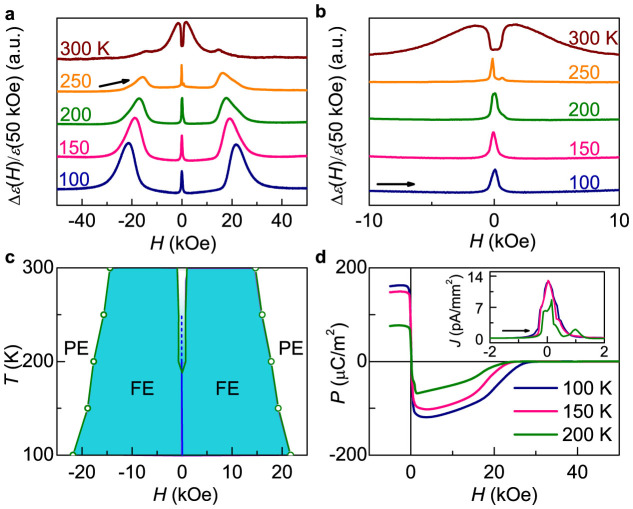
Magnetic field control of electric polarization. (a) The magnetodielectric ratio Δ*ε*(*H*)/*ε*(50 kOe) = [*ε*(*H*)-*ε*(5 kOe)]/*ε*(5 kOe) at selected temperatures. (b) The details of the magnetodielectric behavior around zero field. (c) The magnetoelectric phase diagram of BaSrCoZnFe_11_AlO_22_. (d) Magnetic field reversal of in-plane electric polarization at 100, 150, and 200 K. The inset shows the magnetoelectric current near zero magnetic field.

To check the nature of magnetic-order-induced FE phase, we measured the in-plane (*H* // [100] and *E* // [120]) magnetodielectric properties at different temperatures. Fig. 2a shows the relative change of dielectric constant, Δ*ε*(*H*)/*ε*(50 kOe) = [*ε*(*H*)-*ε*(50 kOe)]/*ε*(50 kOe), at selected temperatures. At high fields, there are broad peaks at all temperatures, corresponding to the transitions from PE to FE or FE to PE phase. Fig. 2b shows the detailed magnetodielectric behaviors near zero field. Below 200 K, only one single dielectric constant peak appears at low magnetic field, indicating the switching of FE domain, which is also confirmed by the *P* – *H* curves in Fig. 2d. At 200 K, a slight shoulder feature starts appearing at finite *H*, signaling a new phase coexists with FE phase near zero field. This new phase is none other than PE phase mentioned above. With further rising temperature, the intensity of the shoulders increase gradually and the height of zero-field single peak decreases simultaneously. At 300 K, the zero-field single peak completely disappears and is replaced by the double peaks around *H* = ±2 kOe, which marks FE – PE – FE double phase transitions. All these magnetodielectric behaviors are in accordance with the *M* – *T* curve (Fig. 1c). Based on the magnetodielectric data, we obtain the magnetoelectric phase diagram shown in Fig. 2c. Below *T*_1_, only transverse cone (FE phase) exists around zero fields and the reversal of electric polarization by magnetic field can be attributed to direct in-plane reversal of the transverse cone state. Above *T*_1_, the PE phase would emerge and coexist with the FE phase near zero fields. With temperature further rising, the PE phase gradually dominates near zero fields at high temperatures.

We also measured the magnetoelectric current (*J*_ME_) below *T*_1_ and integrated it by time to obtain electric polarization (*P*), as displayed in Fig. 2d. The *P* can be reversed by a small *H*. The ME coefficients (*α_h_* = *∂P*/*∂H*) calculated by the magnetoelectric current reach a maximum of ~6000 ps/m at 100 and 150 K, and ~4000 ps/m at 200 K. Above 200 K, because of the low resistivity of our crystal at high temperature, we are not able to pole the sample to obtain reliable ME current. The inset of Fig. 2d shows the *J*_ME_ around zero magnetic fields. At 200 K, besides the current peak at zero field like those at 100 K and 150 K,there is a small current peak around *H* = 1 kOe corresponding to the magnetodielectric shoulder. Therefore, we can attribute the weak current peak to the transition of PE to FE, which suggests chirality is unchanged during the transverse cone - longitudinal cone - transverse cone transitions, similar to that reported in Ba_2_Mg_2_Fe_12_O_22_ (Ref. 21). Moreover, the little current peak also verifies the existence of PE phase near zero magnetic field.

### Nonvolatile electric control of magnetization

We then focus on the converse ME effect in the BaSrCoZnFe_11_AlO_22_ single crystal sample. For multiferroics, there usually exist four kinds of compound domains signified by magnetic and electric order parameters ±*M* and ±*P*^33^. In Y-type hexaferrite, the in-plane *P* is caused by long range transverse conical magnetic order with wave vector along *c* axis, which is in accordance with the spin-current (KNB) model. Therefore, the directions of *P* and *M* of compound domain are totally dependent on the electric field *E* and magnetic field *H* during poling process. To achieve the converse ME effect as large as possible, the converse ME effects were measured after a ME poling procedure in which *H* was decreased from 50 kOe to 5 kOe under application of *E* = 500 kV/m and then the *E* field was turned off. After that, *H* was set to zero or a low field for the converse ME effect measurement.

**Figure 3:**
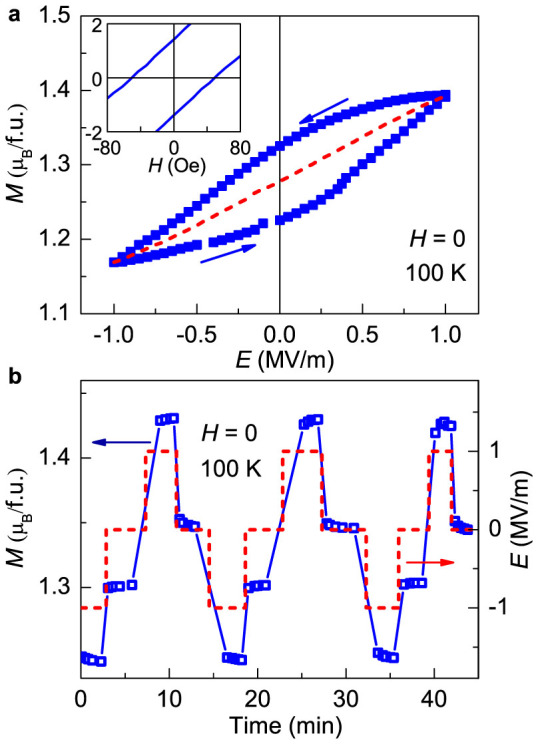
Nonvolatile electric control of magnetization. (a) Magnetization as a function of electric field at 100 K showing the *M − E* hysteresis loop. The inset shows the *M*–*H* hysteresis at low magnetic fields. (b) Four magnetization levels controlled by applying electric field in a repeated sequence of −1 MV/m → 0 → 1 MV/m → 0. No bias magnetic field is needed.

Fig. 3a shows the *E* dependence of *M* along [100] direction at 100 K. The maximum *E* for the converse ME effect measurement is limited under 1 MV/m for safety reason. The substantial hysteresis between the data obtained during increasing and decreasing *E* scan produces a well-defined *M* – *E* hysteresis loop. To estimate the converse ME coefficients in the *M-E* hysteresis case, the magnetizations of the increasing and decreasing *E*-scan data were averaged. A quadratic function: 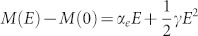 is used to approximate the *E* dependence of *M*, where it includes linear and quadratic *E* terms^25^. The linear coefficient *α_e_* = 3100 ps/m and quadratic coefficient *γ* = 80 ps/MV were obtained, indicating the converse ME effect at 100 K is mainly dominated by the linear term. The smaller difference between the values of *α_e_* and *α_h_* in single crystal than ceramic samples^27^ suggests that the contribution from trapped space charges is effectively suppressed.

The large *M* – *E* hysteresis loop at 100 K indicates its potential for nonvolatile magnetization controlled by *E*, shown in Fig. 3b. After the poling procedure mentioned above, we measured *M* with applying *E* field in a sequence: −1 MV/m – 0 – 1 MV/m – 0 without a magnetic field bias. When applying a negative *E*, the magnetization is reduced owing to the clamped *M* & *P* orders. In contrast, the positive *E* leads to an increase in *M*. It clearly displays that two magnetization values at *E* = 0 are distinctively different after remove *E* = 1 MV/m and *E* = −1 MV/m, due to its large *M* – *E* hysteresis loop shown in Fig. 3a. Moreover, no obvious damping of magnetization was observed after several periods, though we did not apply large enough *E* to make the magnetization saturated.

### The reversal of magnetization by electric field

**Figure 4:**
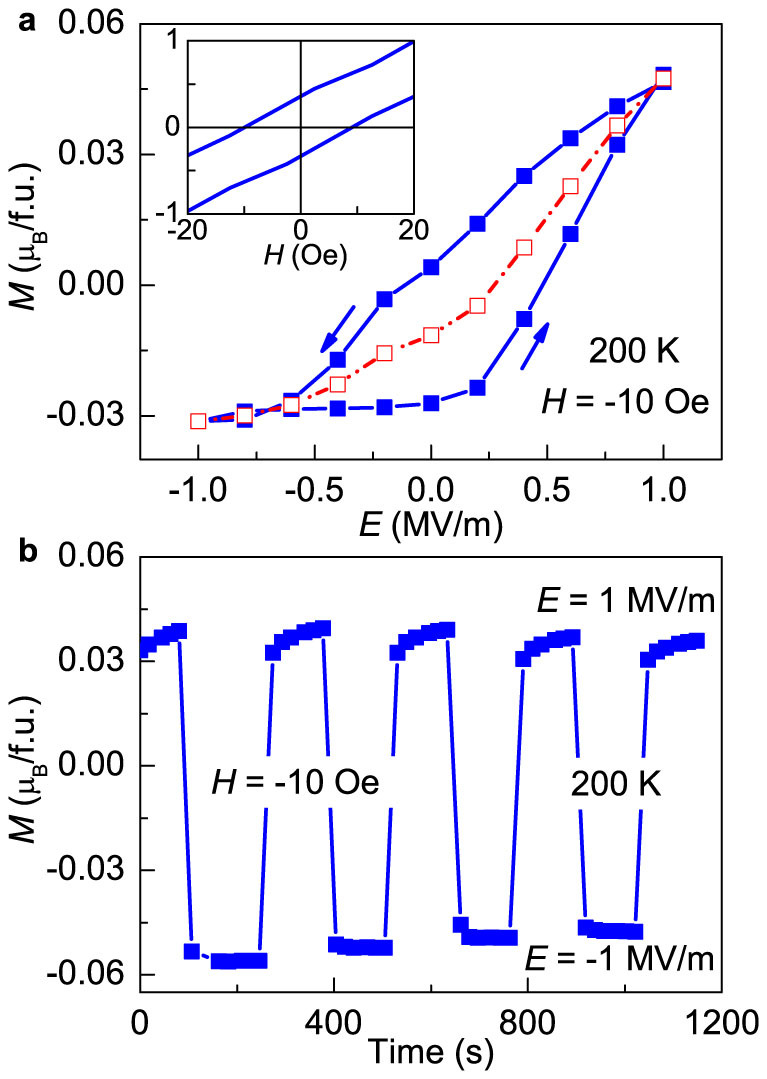
The reversal of magnetization by electric field. (a) Magnetization as a function of electric field measured with a bias magnetic field *H* = −10 Oe at 200 K. The inset shows the *M* – *H* hysteresis loop at low magnetic fields. (b) Electric-field reversal of magnetization under a small bias magnetic field *H* = −10 Oe.

Next, we performed similar measurements at 200 K. Fig. 4a displays the *M* – *E* curve showing a large hysteresis at 200 K with external *H* = −10 Oe along [100] direction. Compared with that at 100 K, the *M* – *E* curve at 200 K is more asymmetric and *M* almost remains constant with decreasing negative *E*-scan. With the same fitting method taken at 100 K, we got *α_e_* = 1100 ps/m and *γ* = 535 ps/MV at 200 K. Such a large quadratic coefficient *γ* is likely to be the reason for the asymmetric shape of the *M* – *E* curve at 200 K. This quadratic converse ME effect is possibly introduced by the new PE phase around zero magnetic field, as our magnetic and magnetodielectric measurements have suggested the existence of mixed phases at this temperature and the transverse cone is responsible for the linear converse ME effect, which has been evidenced by the results at 100 K. the substantial hysteresis at 200 K suggests its potential for nonvolatile magnetic states controlled by *E*, just like that at 100 K. A distinguished feature at 200 K is the reversal of *M* by *E* field. Fig. 4b demonstrates the repeated reversal of *M* by switching the polarity of electric field (*E* = ±1 MV/m) under a small bias *H* = −10 Oe at 200 K. The positive *E* causes an increase in *M*, while the negative *E* leads to polarity reversal of *M*.

## Discussion

The electric polarization in Y-type hexaferrite can be well explained by the spin-current model ***P*** ∝***k***_0_ × (***S****_i_* × ***S****_j_*), where ***S_i_*** and ***S_j_*** denote the neighbor spins at site *i* and *j*, ***k_0_*** // [001] is the magnetic propagation vector. According to this model, the screw or longitudinal conical spin configurations cannot generate ***P*** due to spin chirality Σ(***S****_i_* × ***S****_j_*) // ***k***_0_. However, the transverse cone is a FE phase. Below 200 K, the transverse cone (FE) still persists after we decrease *H* from high field to zero, which is responsible for the direct reversal of *P* by *H* and the linear converse ME effect. In benefit of the large converse ME susceptibility (α*_e_* = 3100 ps/m) and substantial *M-E* hysteresis loop, we achieve nonvolatile distinguished change of *M* by *E*, that is to say, the magnetizations differ even after removing +*E* and −*E*. This result provides the potential for future nonvolatile magnetic memory device controlled by *E*.

Due to the clamped *M* & *P* orders in multiferroic hexaferrites, there are only two kinds of compound domains: +*M*/+*P* and −*M*/−*P* since +*E* and +*H* are applied during poling process. The manipulation of *M* by *E* can be thought as the result of the compound domain wall movement. Like the hysteresis loop in the *M* – *H* loop, the hysteresis characteristics for *M* – *E* curves appear due to resistance of domain wall movement. The relatively large coercive field (*H*_c_ = 50 Oe) in the *M* – *H* loop is probably responsible for the substantial *M* – *E* hysteresis loop, which is the prerequisite for nonvolatile magnetization controlled by *E*.

Different from the case at 100 K where the linear converse ME effect dominates, the contribution of quadratic converse ME effect is no longer negligible at 200 K. Magnetodielectric and *J*_ME_ measurements at 200 K suggest that PE and FE phase coexist at zero field, that is to say, besides the transverse cone phase, there is a longitudinal cone or proper screw phase when we ramp from high-*H* down to zero. Thus, it could be the existence of the PE phase (proper screw or longitudinal cone) responsible for the quadratic converse ME effect.

In summary, we have demonstrated pronounced ME effects in the Y-type hexaferrite BaSrCoZnFe_11_AlO_22_ single crystal at different temperatures. Its direct ME susceptibility can reach as high as 6000 ps/m at 100 K, and 4000 ps/m at 200 K. In benefit of the large *M* – *E* hysteresis loop as well as the large converse ME coefficients (3100 and 1100 ps/m at 100 and 200 K, respectively) in single crystals, nonvolatile *E* control of magnetization can be realized. In addition, reversal of magnetization by *E* field was also demonstrated at 200 K. These excellent properties promise its potential for the future magnetoelectric functional devices. Further tailoring of the composition of Y-type hexaferrites may eventually realize these diverse ME effects at room temperature.

## Methods

### Sample preparation

Single-crystal samples of the Y-type hexaferrite BaSrCoZnFe_11_AlO_22_ were prepared by Na_2_O-Fe_2_O_3_ flux method in air as described elsewhere^34^. The as-grown samples were annealed at 900°C in O_2_ atmosphere to enhance the resistivity^35^.

### Electric and magnetic measurements

For electrical measurements, the crystals were cut into thin plates with the widest faces perpendicular to the [120] direction in the hexagonal setting and then were painted with sliver paste on the widest faces. The *J*_ME_ and dielectric constant were measured by an electrometer (Keithley 6517B) and a LCR meter (Aglient, 4980A), respectively, in a Cryogen-free Superconducting Magnet System (Oxford Instruments, TeslatronPT). The converse ME effects were measured by using a magnetometer with a homemade sample holder (MPMS, Quantum Design). Before *J*_ME_ measurements, we need to carry out the following steps to pole our sample: (1) *E* = 500 kV/m was applied at *H* = 50 kOe along [100] direction where sample was in a high-field PE phase; (2) ramped down the *H* to 5 kOe, where the sample was driven to the intermediate field FE phase, then withdrew electric field and shortened two electrodes for 30 mins to release free charges; (3) swept magnetic field with 25 Oe/s to −50 kOe and measured the *J*_ME_.

## Author Contributions

Y.S. and S.S conceived the project. S.S prepared the samples and performed the experiments. Y.C. helped in experiments and analysis of the data. All authors contributed to writing the paper.
